# Dental Commencement of Meharry Medical College

**Published:** 1893-04

**Authors:** 


DENTAL COMMENCEMENT OF MEHARRY MEDICAL COLLEGE.—The seventh annual commencement of the Meharry Dental Department, of Central Tennessee College, Nashville, Tenn., was held in connection with that of the Medical Department., February 7th, at the Gospel Tabernacle, in the presence of an audience of over three thousand people.

S. R. Thompson, of Tennessee, and A. M. Wilkins, of Georgia, were the graduates in dentistry; the valedictory address being delivered by the latter.

Seven students have been enrolled during this session.

This is the only institution in the Southern States for the education of colored dentists. The graduates of former years from this school have been well received by the white dentists of the South, and have been well patronized by their own people.

